# Supplementary material for: A method to estimate the contribution of regional genetic associations to complex traits from summary association statistics
Source: Sci Rep. 2016 Jun 8;6:27644. doi: 10.1038/srep27644 (PMC4897708; doi:10.1038/srep27644)
Supplement: Supplementary Information [file srep27644-s1.pdf]

Title: A method to estimate the contribution of regional genetic associations to complex traits from summary association statistics

Guillaume Pare,[1,2,3,4,*] Shihong Mao,[3] Wei Q. Deng[5]

1 Department of Pathology and Molecular Medicine, McMaster University, Hamilton, ON L8S 4L8, Canada, 2 Population Genomics Program, Department of Clinical Epidemiology and Biostatistics, McMaster University, Hamilton, ON L8S 4L8, Canada, 3 Population Health Research Institute, Hamilton Health Sciences and McMaster University, Hamilton, ON L8L 2X2, Canada, 4 Thrombosis and Atherosclerosis Research Institute, Hamilton, ON L8L 2X2, Canada, 5 Department of Statistical Sciences, University of Toronto, Toronto, ON M5S 3G3, Canada
*Corresponding author: pareg@mcmaster.ca

Supplemental information:

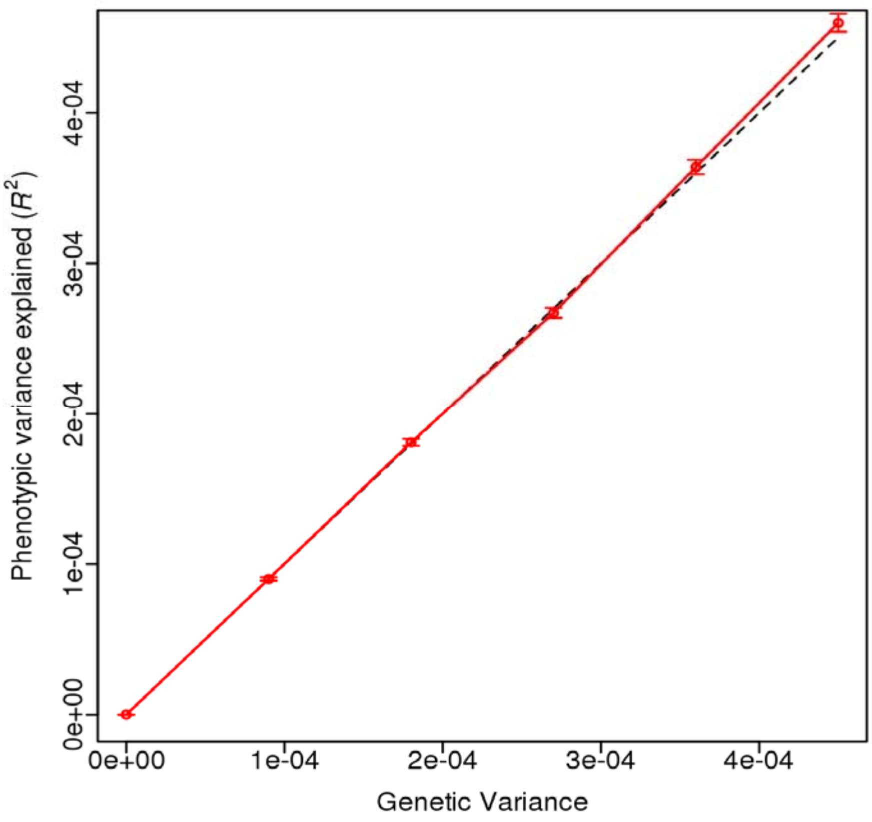

Figure S1: Regional genetic variance estimated by LD Adjusted Regional Genetic Variance (LARGV) as a function of true genetic variance in data simulated from the 1000G project.

5,000 individuals were simulated for 450 contiguous SNPs using phased haplotypes from 1000G. The genetic effect of each SNP was randomly selected from a normal distribution according to a pre-defined, unobserved, true regional genetic variance selected assuming genome-wide heritability varying from 0 to 0.5. For each regional genetic variance set-point, 1,000 simulations were completed and the average (±SD) regional genetic variance estimated by LARGV illustrated. The dashed black line represents the identity line (y = x).

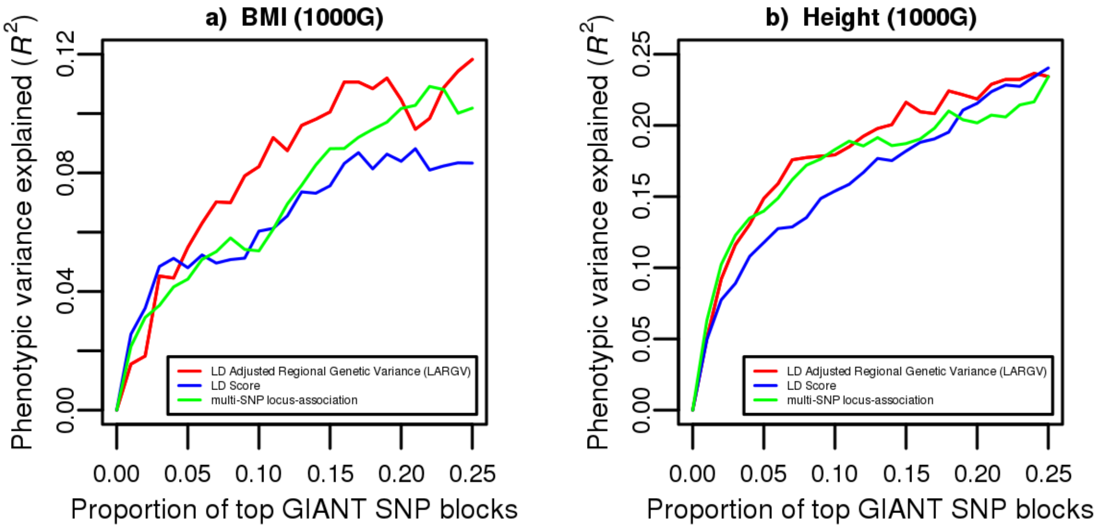

Figure S2: Genetic variance as a function of proportion of top SNP blocks using LD data from 1000G project European participants

Genetic variance in HRS based on SNP block ranking derived from GIANT summary association statistics using LD data from 1000G project European participants. Three methods were tested to rank SNP blocks: LARGV (red), LD Score (blue) and multi-SNP locus-association (green). The median SNP block size was 250 Kb (i.e. 85-95 SNPs). Genetic variance was calculated in HRS using variance component models for BMI (Panel A) and height (Panel B).

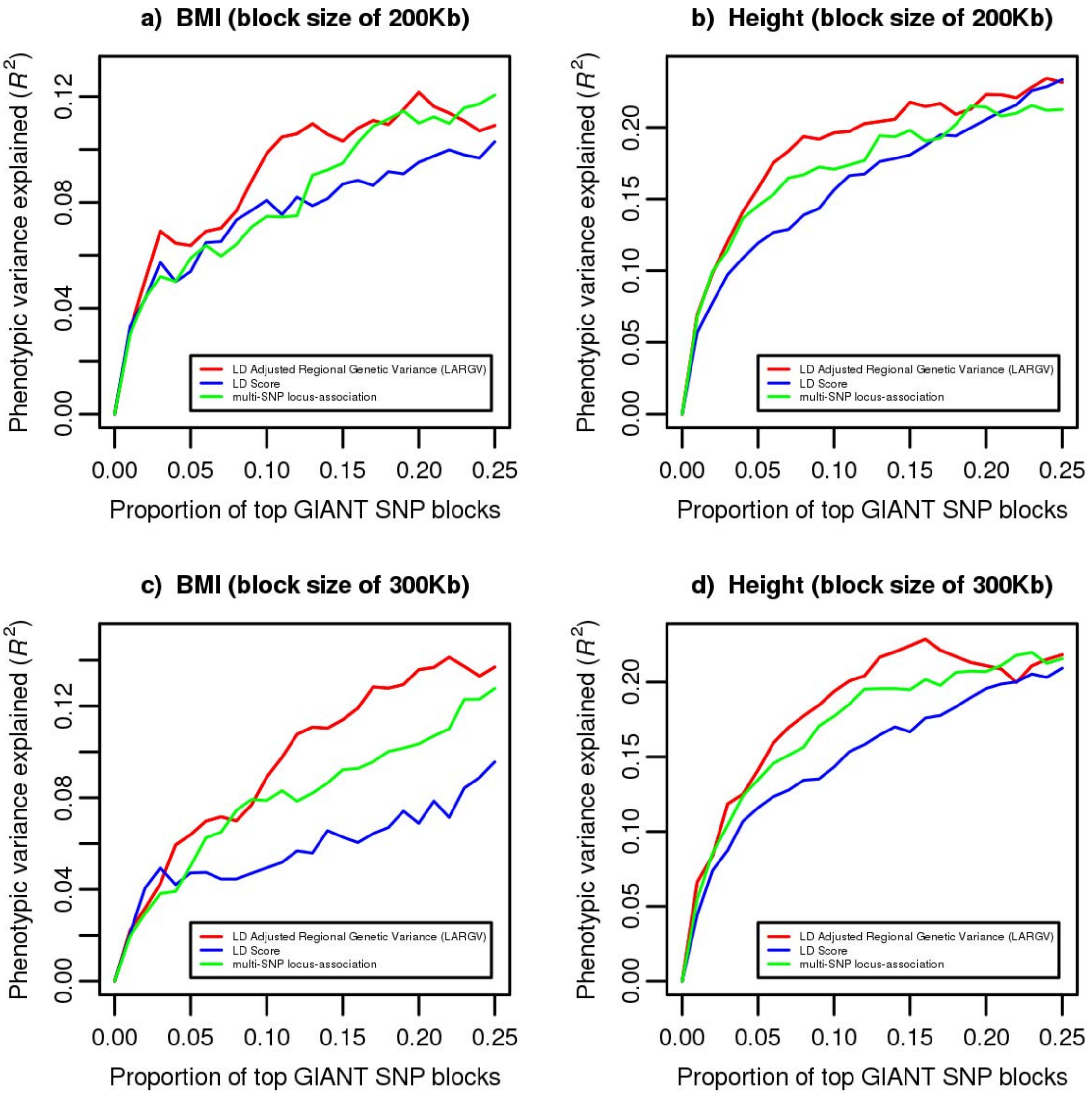

Figure S3: Genetic variance as a function of proportion of top SNP blocks when varying SNP block size

Genetic variance in HRS based on SNP block ranking derived from GIANT summary association statistics. Three methods were tested to rank SNP blocks: LARGV (red), LD Score (blue) and multi-SNP locus-association (green). The median SNP block size was 200

Kb (panels A and B) or 300 Kb (panels C and D). Genetic variance was calculated in HRS using variance component models for BMI (panels A and C) and height (panels B and D).

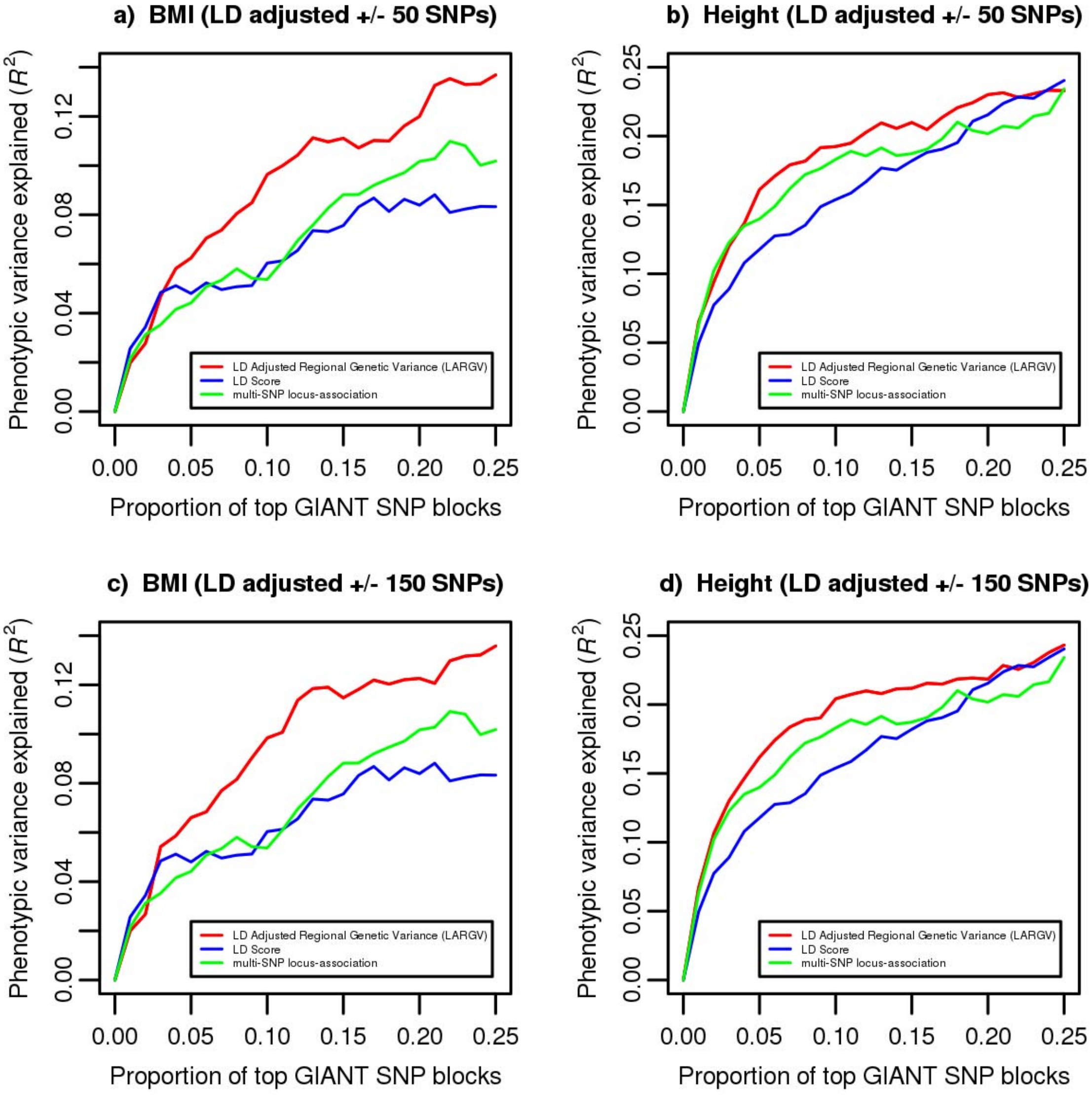

Figure S4: Genetic variance as a function of proportion of top SNP blocks when varying the number of neighboring SNPs included in LD calculations

Genetic variance in HRS based on SNP block ranking derived from GIANT summary association statistics. Three methods were tested to rank SNP blocks: LARGV (red), LD Score (blue) and multi-SNP locus-association (green). The median SNP block size was 250 Kb. The number of neighboring SNPs included in LD calculations was +/- 50 SNPs (panels

A and B) or +/- 150 SNPs (panels C and D). Genetic variance was calculated in HRS using variance component models for BMI (panels A and C) and height (panels B and D).
